# Supplementary material for: Susceptibility to positive versus negative emotional contagion: First evidence on their distinction using a balanced self-report measure
Source: PLoS One. 2024 May 14;19(5):e0302890. doi: 10.1371/journal.pone.0302890 (PMC11093349; doi:10.1371/journal.pone.0302890)
Supplement: S1 Appendix — This review strives to (1) give a systematic overview of existing self-report instruments to measure individuals’ susceptibility to emotional contagion (SEC), (2) to describe the theoretical framework of existing scales, and (3) to review the specific items that are used to assess individuals’ SEC in different scales. (DOCX) [file pone.0302890.s001.docx]

**S1 Appendix. Systematic review of published self-report measures of susceptibility to emotional contagion.**

This review strives to (1) give a systematic overview of existing self-report instruments to measure individuals’ susceptibility to emotional contagion (SEC), (2) to describe the theoretical framework of existing scales, and (3) to review the specific items that are used to assess individuals’ SEC in different scales.

**Method**

***Literature search***

The methods to identify potentially relevant publications included (1) searching multiple bibliographic databases (PsycINFO, PSYNDEX, PubMed, ERIC), (2) scanning reference lists of existing reviews and eligible studies, (3) contacting scholars in the area of research, and (4) broadly searching the internet and academic online networks (www.researchgate.net, www.academia.edu, www.semanticscholar.org). Because different names are commonly used for similar phenomena of EC, a variety of search terms were used, including all possible combinations of the terms “emotional”, “emotions”, “contagion”, “contagious”, “transmission”, “transfer”, "crossover", “susceptibility”, “measurement”, “measure”, “scale”, “questionnaire”, “index”, and “self-report”.

***Inclusion criteria***

Following this extensive literature search, the following inclusion criteria were used to select publications for further analyses: First, at least one of the items used in the measure was related to the phenomenon of SEC. Second, the focus of the publication lies on either scale development or scale validation and/or the reported measure could be and is intended to be used beyond a single study (e.g. included reports on content validity, construct validity, criterion validity, or norms, or instructions on how to use the measure, calculate scores, and/or what specific items are used). There were no constraints regarding the publication date, the country of origin or the language of the publication.

***Data extraction and analysis***

Subsequently, the selected publications were reviewed and analyzed regarding (1) their year of development or publication, (2) target group(s), (3) theoretical conceptualization and framework, (4) subscales, (5) response scale, and (6) the number of items related to SEC in total, SEC of positive emotions, SEC of negative emotions, and general SEC.

**Results & Discussion**

***Overview***

In total, we found 102 publications that matched our previously defined search criteria. Of these publications, 28 fulfilled the inclusion criteria for further analyses and 74 were excluded (for a complete list of the included measures and an overview of their characteristics, see Table S1.1). These publications were excluded either because they reported on measures other than self- or other-report measures (*n* = 5; e.g., picture-based tests or interviews), the specific items were not available (*n* = 20), the authors used translated or adapted versions of already included measures (*n* = 34), or none of the items were related to SEC (*n* = 15).

The included publications were published between 1972 and 2019 and all of them either aimed at the development and/or validation of a self-/other report measure. Their main target population were adults (*n* = 21), followed by adolescents (*n* = 4) and children (*n* = 3) with one of them targeting both children and adolescents and one of them adults and adolescents, respectively. Of the included measures, 26 were self-report measures and two were parent-report measures.

***Self-report measures of SEC***

Of the included measures, 24 focused on empathy (including specialized constructs like empathic drive and positive empathy), one focused on relationship quality, one on emotional sensitivity, one on vicarious distress, and only one of the reviewed measures focused explicitly on SEC. Overall, seven of the included measures were developed as unidimensional measures and 21 of them entailed two or more subscales. Of these 21 bi-/multidimensional measures, six included a subscale explicitly addressing SEC (labeled either “Susceptibility to Emotional Contagion” or “Emotional Contagion”). Taken together, while all of the included measures contained at least one item related to SEC, there were only seven scales or subscales explicitly addressing SEC. These measures were the Questionnaire Measure of Emotional Empathy (QMEE; Mehrabian & Epstein, 1972), the Emotional Contagion Scale (ECS; Doherty, 1997), the Multidimensional Emotional Empathy Scale (MDEES; Caruso & Mayer, 1998), the Empathy Questionnaire (EmQue; Rieffe et al., 2010), the Questionnaire of Cognitive and Affective Empathy (QCAE; Reniers et al., 2011), the Basic Empathy Scale – Adults (BES-A; Carré et al., 2013), and the Multidimensional Empathy Questionnaire for Children (MEQC; Richaud et al., 2017).

***Theoretical conceptualization and framework***

Most of the measures that explicitly address SEC were originally developed to assess empathy (*n* = 6) and they refer to a theoretical conceptualization of empathy with two distinct components: A cognitive empathy component and an affective empathy component. Within this model, cognitive empathy represents an understanding of other people’s experiences and affective empathy refers to the ability to vicariously experience the emotional experiences of other individuals (e.g. Decety & Jackson, 2004). Despite this definition of empathy as an ability to take perspective and "feel with others", some affective empathy scales in fact address the concept of SEC in terms of a tendency to automatically und unconsiously feel what others are feeling. Intriguingly, while these measures actually aim to assess SEC, their scales/subscales are often misleadingly labeled "emotional contagion" (Carré et al. , 2013; Caruso & Mayer, 1998; Reniers et al., 2011; Richaud et al., 2017; Rieffe et al., 2010). In conclusion, (1) most of the published measures of SEC originally aim to measure empathy and (2) their SEC scales are partly mislabeled.

***Items assessing SEC***

While only seven measures explicitly addressed SEC either as a unidimensional measure or as a subscale of a measure, all of the included measures contained at least one item related to SEC. The total number of items in all included measures was 720 and ranged from 5 to 64 items per measure (*M* = 25.7 items, *SD* = 15.1 items). Of these items, we identified 132 items that were at least tangentially related to the SEC of positive or negative emotions or to a general SEC with no clear valence (summarized in Table S1.2). Of these identified items, 71 were related to the SEC of negative emotions (53.8 % of SEC related items), 34 to the SEC of positive emotions (25.8 %), and 27 to a general SEC (20.5 %).

In the subgroup of measures explicitly focusing on SEC (7 measures), there were 164 items in total, of which we identified 50 items to directly addressed SEC (30.5%). Of these items that were related to SEC, 12 were related to positive SEC (24% of all items related to SEC), 30 were related to negative SEC (60%), and eight to general SEC (16%). In conclusion, the majority of items related to SEC focus on negative SEC and, in the existing measures of SEC, positive SEC is not equally represented.

***Conclusion and implications***

First, there seem to be only very few existing scales that address the measurement of individuals’ SEC. Second, most of these measures originally aim to assess different components of empathy and SEC is conceptualized as part of the affective empathy component. Third, these existing self-report measures of SEC have a striking bias on the transmission of negative emotions while the transmission of positive emotions has not been equally represented.

**References**

Baron-Cohen, S., & Wheelwright, S. (2004). The Empathy Quotient: An investigation of adults with asperger syndrome or high functioning autism, and normal sex differences. *Journal of Autism and Developmental Disorders*, *34*(2), 163–175. doi:10.1023/B:JADD.0000022607.19833.00

Batchelder, L., Brosnan, M., & Ashwin, C. (2017). The development and validation of the Empathy Components Questionnaire (ECQ). *PLOS ONE*, *12*(1), e0169185. doi:10.1371/journal.pone.0169185

Bryant, B. K. (1982). An index of empathy for children and adolescents. *Child Development*, *53*(2), 413–425. doi:10.2307/1128984

Carré, A., Stefaniak, N., D’Ambrosio, F., Bensalah, L., & Besche-Richard, C. (2013). The Basic Empathy Scale in adults (BES-A): Factor structure of a revised form. *Psychological Assessment*, *25*(3), 679–691. doi:10.1037/a0032297

Caruso, D. R., & Mayer, J. D. (1998). *A measure of emotional empathy for adolescents and adults*. Unpublished Manuscript. Retrieved from https://mypages.unh.edu/sites/default/files/jdmayer/files/empathy_article_2000.pdf

Cramer, D. (1986). An item factor analysis of the revised Barrett-Lennard Relationship Inventory. *British Journal of Guidance & Counselling*, *14*(3), 314–325. doi:10.1080/03069888608253521

Dadds, M. R., Hunter, K., Hawes, D. J., Frost, A. D. J., Vassallo, S., Bunn, P., Merz, S., & Masry, Y. E. (2008). A measure of cognitive and affective empathy in children using parent ratings. *Child Psychiatry and Human Development*, *39*(2), 111–122. doi:10.1007/s10578-007-0075-4

Davis, M. H. (1983). Measuring individual differences in empathy: Evidence for a multidimensional approach. *Journal of Personality and Social Psychology*, *44*(1), 113–126. doi:10.1037/0022-3514.44.1.113

Decety, J., & Jackson, P. L. (2004). The functional architecture of human empathy. *Behavioral and Cognitive Neuroscience Reviews*, *3*(2), 71–100. doi:10.1177/1534582304267187

Doherty, R. W. (1997). The emotional contagion scale: A measure of individual differences. *Journal of Nonverbal Behavior*, *21*(2), 131–154. doi:10.1023/A:1024956003661

Garton, A. F., & Gringart, E. (2005). The development of a scale to measure empathy in 8-and 9-year old children. *Australian Journal of Educational & Developmental Psychology*, *5*, 17–25.

Guarino, L. R. (2003). *Emotional sensitivity: A new measure of emotional lability and its moderating role in the stress-illness relationship* (Doctoral Dissertation, University of York). Retrieved from http://etheses.whiterose.ac.uk/9899/1/434026.pdf

Grynberg, D., Heeren, A., & Luminet, O. (2012). Development and validation of the Vicarious Distress Questionnaire. *Canadian Journal of Behavioural Science / Revue Canadienne Des Sciences Du Comportement*, *44*(2), 138–145. doi:10.1037/a0027509

Heynen, E. J. E., Helm, G. H. P. V., Stams, G. J. J. M., & Korebrits, A. M. (2016). Measuring empathy in a German youth prison: A validation of the German version of the Basic Empathy Scale (BES) in a sample of incarcerated juvenile offenders. *Journal of Forensic Psychology Practice*, *16*(5), 336–346. doi:10.1080/15228932.2016.1219217

Hojat, M., Gonnella, J. S., Nasca, T. J., Mangione, S., Veloksi, J. J., & Magee, M. (2002). The Jefferson Scale of Physician Empathy: Further psychometric data and differences by gender and specialty at item level. *Academic Medicine: Journal of the Association of American Medical Colleges*, *77*(10 Suppl), S58-60. doi:10.1097/00001888-200210001-00019

Innamorati, M., Ebisch, S. J. H., Gallese, V., & Saggino, A. (2019). A bidimensional measure of empathy: Empathic Experience Scale. *PLOS ONE*, *14*(4), e0216164. doi:10.1371/journal.pone.0216164

Jordan, M. R., Amir, D., & Bloom, P. (2016). Are empathy and concern psychologically distinct? *Emotion*, *16*(8), 1107–1116. doi:10.1037/emo0000228

Karlstetter, W. (2017). Emotional Empathic Drive Short Scale (EED). *Zusammenstellung sozialwissenschaftlicher Items und Skalen (ZIS).* doi:10.6102/zis250 Retrieved from https://zis.gesis.org/skala/Karlstetter-Emotional-Empathic-Drive-Short-Scale-(EED)

Light, S. N., Moran, Z. D., Zahn-Waxler, C., & Davidson, R. J. (2019). The measurement of positive valence forms of empathy and their relation to anhedonia and other depressive symptomatology. *Frontiers in Psychology*, *10*. doi:10.3389/fpsyg.2019.00815

Mehrabian, A., & Epstein, N. (1972). A measure of emotional empathy. *Journal of Personality*, *40*(4), 525–543. doi:10.1111/j.1467-6494.1972.tb00078.x

Olderbak, S., Sassenrath, C., Keller, J., & Wilhelm, O. (2014). An emotion-differentiated perspective on empathy with the emotion specific empathy questionnaire. *Frontiers in Psychology*, *5*. doi:10.3389/fpsyg.2014.00653

Reniers, R. L. E. P., Corcoran, R., Drake, R., Shryane, N. M., & Völlm, B. A. (2011). The QCAE: A Questionnaire of Cognitive and Affective Empathy. *Journal of Personality Assessment*, *93*(1), 84–95. doi:10.1080/00223891.2010.528484

Richaud, M. C., Lemos, V. N., Mesurado, B., & Oros, L. (2017). Construct validity and reliability of a new spanish empathy questionnaire for children and early adolescents. *Frontiers in Psychology*, *8*. doi:10.3389/fpsyg.2017.00979

Rieffe, C., Ketelaar, L., & Wiefferink, C. H. (2010). Assessing empathy in young children: Construction and validation of an Empathy Questionnaire (EmQue). *Personality and Individual Differences*, *49*(5), 362–367. doi:10.1016/j.paid.2010.03.046

Shen, L. (2010). On a scale of state empathy during message processing. *Western Journal of Communication*, *74*(5), 504–524. doi:10.1080/10570314.2010.512278

Spreng, R. N., McKinnon, M. C., Mar, R. A., & Levine, B. (2009). The Toronto Empathy Questionnaire: Scale development and initial validation of a factor-analytic solution to multiple empathy measures. *Journal of Personality Assessment*, *91*(1), 62–71. doi:10.1080/00223890802484381

Suzuki, Y., & Kino, K. (2008). Development of the Multidimensional Empathy Scale (MES): Focusing on the distinction between self- and other-orientation. *Japanese Journal of Educational Psychology*, *56*, 487–497. doi:10.5926/jjep1953.56.4_487

Vachon, D. D., & Lynam, D. R. (2016). Fixing the problem with empathy: Development and validation of the affective and cognitive measure of empathy. *Assessment*, *23*(2), 135–149. doi:10.1177/1073191114567941

Vossen, H. G. M., Piotrowski, J. T., & Valkenburg, P. M. (2015). Development of the Adolescent Measure of Empathy and Sympathy (AMES). *Personality and Individual Differences*, *74*, 66–71. doi:10.1016/j.paid.2014.09.040

**Table S1.1**

Overview of All Measures Included in the Systematic Review

| Measure | References | Report form | Target group | Language | *N* items | Underlying construct | Scales |
| --- | --- | --- | --- | --- | --- | --- | --- |
| Questionnaire Measure of Emotional Empathy (QMEE) | Mehrabian & Epstein (1972) | Self-report | Adults | English | 33 | Empathy | Susceptibility to emotional contagion;  Appreciation of the feelings of unfamiliar and distant others;  Extreme emotional responsiveness;  Tendency to be moved by others positive emotional experiences;  Tendency to be moved by others negative emotional experiences;  Sympathetic tendency;  Willingness to be in contact with others who have problems |
| Index of Empathy for Children and Adolescents (IECA) | Bryant (1982) | Self-report | Children, adolescents | English | 22 | Empathy | none |
| Interpersonal Reactivity Index (IRI) | Davis (1983) | Self-Report | Adults | English | 28 | Empathy | Perspective-taking;  Fantasy; Personal distress; Empathic concern |
|  |  |  |  |  |  |  | *(continued)* |

| Table S1.1 (continued) | | | | | | | |
| --- | --- | --- | --- | --- | --- | --- | --- |
| Measure | References | Report form | Target group | Language | *N* items | Underlying construct | Scales |
| Revised Barrett-Lennard Relationship Inventory (RI) | Cramer (1986) | Self-Report | Adults | English | 64 | Relationship quality | Level of regard; Advice-giving; Empathy; Unconditionality of regard; Congruence |
| Emotional Contagion Scale (ECS) | Doherty (1997) | Self-Report | Adults | English | 15 | Susceptibility to Emotional Contagion | none |
| Multidimensional Emotional Empathy Scale (MDEES) | Caruso & Mayer (1998) | Self-Report | Adolescents, adults | English | 30 | Empathy | Empathic suffering; Positive sharing; Responsive crying; Emotional attention; Feeling for others; Emotional contagion |
| Jefferson Scale of Physician Empathy (JSPE) | Hojat et al. (2002) | Self-Report | Adults (physicians) | English | 20 | Empathy | none |
| Emotional Sensitivity Scale (ESS) | Guarino (2003) | Self-Report | Adults | English | 41 | Emotional sensitivity | none |
| Empathy Quotient (EQ) | Baron-Cohen & Wheelwright (2004) | Self-Report | Adults (asperger syndrome) | English | 60 | Empathy | none (20 filler items) |
| Feeling and Thinking Scale (FTS) | Garton & Gringart (2005) | Self-Report | Children | English | 12 | Empathy | Affective empathy;  Cognitive empathy |
| Griffith Empathy Measure (GEM) | Dadds et al. (2008) | Parent-Report | Parents | English | 23 | Empathy | Affective empathy;  Cognitive empathy |
|  |  |  |  |  |  |  | *(continued)* |

| Table S1.1 (continued) |  |  |  |  |  |  |  |
| --- | --- | --- | --- | --- | --- | --- | --- |
| Measure | References | Report form | Target group | Language | *N* items | Underlying construct | Scales |
| Multidimensional Empathy Scale (MES) | Suzuki & Kino (2008) | Self-Report | Adults | Japanese | 24 | Empathy | Other-oriented emotional reactivity; Self-oriented emotional reactivity; Emotional susceptibility; Perspective taking; Fantasy |
| Toronto Empathy Questionnaire (TEQ) | Spreng et al. (2009) | Self-Report | Adults | English | 16 | Empathy | none |
| Empathy Questionnaire (EmQue) | Rieffe et al. (2010) | Parent-Report | Parents | English | 20 | Empathy | Emotional contagion; Attention to others' feelings; Prosocial actions |
| State Empathy Scale (SES) | Shen (2010) | Self-Report | Adults | English | 16 | Empathy | Affective empathy; Cognitive empathy; Associative empathy |
| Questionnaire of Cognitive and Affective Empathy (QCAE) | Reniers et al. (2011) | Self-Report | Adults | English | 31 | Empathy | Cognitive empathy (perspective taking, online simulation); Affective empathy (emotion contagion, proximal responsivity, peripheral responsivity) |
| Vicarious Distress Questionnaire (VDQ) | Grynberg et al. (2012) | Self-Report | Adults | English | 18 | Vicarious distress | Distress; Avoidance;  Support |
| Basic Empathy Scale – Adults (BES-A) | Carré et al. (2013) | Self-Report | Adults | English | 20 | Empathy | Emotional disconnection; Emotional contagion; Cognitive empathy |
|  |  |  |  |  |  |  | *(continued)* |

| Table S1.1 (continued) |  |  |  |  |  |  |  |
| --- | --- | --- | --- | --- | --- | --- | --- |
| Measure | References | Report form | Target group | Language | *N* items | Underlying construct | Scales |
| Emotion-Specific Empathy Questionnaire (ESEQ) | Olderbak et al. (2014) | Self-Report | Adults | English | 60 | Empathy | Anger affective empathy; Anger cognitive empathy; Disgust affective empathy; Disgust cognitive empathy; Fear affective empathy; Fear cognitive empathy; Happy affective empathy; Happy cognitive empathy; Sad affective empathy; Sad cognitive empathy; Surprise affective empathy; Surprise cognitive empathy |
| Adolescent Measure of Empathy and Sympathy (AMES) | Vossen et al. (2015) | Self-Report | Adolescents | English | 12 | Empathy, sympathy | Affective empathy;  Cognitive empathy;  Sympathy |
| Affective and Cognitive Measure of Empathy (ACME) | Vachon & Lynam (2016) | Self-Report | Adults | English | 36 | Empathy | Cognitive empathy; Affective resonance; Affective dissonance |
| Empathy Index (EI) | Jordan et al. (2016) | Self-Report | Adults | English | 14 | Empathy | Empathy; Behavioral contagion |
| Basic Empathy Scale – Youth (BES-Y) | Heynen et al. (2016) | Self-Report | Adolescents | German, English | 12 | Empathy | Affective empathy; Cognitive empathy |
| Empathy Components Questionnaire (ECQ) | Batchelder et al. (2017) | Self-Report | Adults | English | 28 | Empathy | Affective reactivity; Cognitive drive; Affective ability; Affective drive; Cognitive ability; Social perspective-taking |
|  |  |  |  |  |  |  | *(continued)* |

| Table S1.1 (continued) |  |  |  |  |  |  |  |
| --- | --- | --- | --- | --- | --- | --- | --- |
| Measure | References | Report form | Target group | Language | *N* items | Underlying construct | Scales |
| Emotional Empathic Drive Short Scale (EEDS) | Karlstetter (2017) | Self-Report | Adults | English, German | 5 | Empathic drive | none |
| Multidimensional Empathy Questionnaire for Children (MEQC) | Richaud et al. (2017) | Self-Report | Children | English, Spanish | 15 | Empathy | Emotional contagion; Self-awareness; Perspective-taking; Emotional regulation; Empathic action |
| Empathic Experience Scale (EES) | Innamorati et al. (2019) | Self-Report | Adults | English, Italian | 30 | Empathy | Intuitive understanding; Vicarious experience |
| Positive Empathy Scale (PES) | Light et al. (2019) | Self-Report | Adults | English | 15 | Positive empathy | Empathic happiness; Empathic cheerfulness |

**Table S1.2**

Overview of the Published Items Addressing SEC of Positive and Negative Emotions or a General SEC

| Measure | Items addressing Positive SEC | Items addressing negative SEC | Items addressing SEC with no clear valence |
| --- | --- | --- | --- |
| QMEE (Mehrabian & Epstein, 1972) | Another’s laughter is not catching for me. | I become nervous if others around me seem to be nervous; I don’t get upset just because a friend is acting upset; I am able to remain calm even though those around me worry; I cannot continue to feel OK if people around me are depressed; I often find that I can remain cool in spite of the excitement around me. | The people around me have a great influence on my mood. |
| IRI (Davis, 1983) |  | Being in a tense emotional situation scares me; When I see someone get hurt, I tend to remain calm. |  |
| IECA (Bryant, 1982) | Even when I don't know why someone is laughing, I laugh too. | Seeing a boy/girl who is crying makes me feel like crying; I get upset when I see a boy/girl being hurt. |  |
| RI (Cramer, 1986) |  | When I am hurt or upset he/she can recognize my feelings exactly, without becoming upset her/himself; If I show that I am angry with her/him, he/she becomes hurt or angry with me, too. | Sometimes he/she thinks that I feel a certain way, because that's the way he/she feels. |
|  |  |  | *(continued)* |
| Table S1.2 (continued) |  |  |  |
| Measure | Items addressing Positive SEC | Items addressing negative SEC | Items addressing SEC with no clear valence |
| ECS (Doherty, 1997) | Being with a happy person picks me up when I'm feeling down; When someone smiles warmly at me, I smile back and feel warm inside; Being around happy people fills my mind with happy thoughts. | If someone I'm talking with begins to cry, I get teary-eyed; I clench my jaws and my shoulders get tight when I see the angry faces on the news; I tense when overhearing an angry quarrel. I notice myself getting tense when I'm around people who are stressed out. |  |
| MEES (Caruso & Mayer, 1998) | When I'm with other people who are laughing I join in; I feel happy when I see people laughing and enjoying themselves; If a crowd gets excited about something so do I; Seeing other people smile makes me smile; Being around happy people makes me feel happy, too; I feel other people's joy. | The suffering of others deeply disturbs me; If someone is upset I get upset, too; I feel other people's pain; Being around people who are depressed brings my mood down; It hurts to see another person in pain. | It's easy for me to get carried away by other people's emotions; My feelings are my own and don't reflect how others feel. |
| JSPE (Hojat et al., 2002) |  |  | I do not allow myself to be touched by intense emotional relationships between my patients and their family members. |
| ESS (Guarino, 2003) | I find it easy to share in other happiness. | I get upset when other people are having a hard time. | I'm easily affected by others' emotional problems. |
|  |  |  | *(continued)* |
| Table S1.2 (continued) |  |  |  |
| Measure | Items addressing Positive SEC | Items addressing negative SEC | Items addressing SEC with no clear valence |
| EQ (Baron-Cohen & Wheelwright, 2004) |  | Seeing people cry doesn’t really upset me; I get upset if I see people suffering on news programs. | I can tune into how someone else feels rapidly and intuitively;  I tend to get emotionally involved with a friend’s problems. |
| FTS (Garton & Gringart, 2005) |  | When people around me are nervous or worried, I get a bit scared and worried too; Sometimes I feel helpless when people around me are upset. |  |
| GEM (Dadds et al., 2008) | My child acts happy when another person is acting happy; My child laughs when seeing another child laugh. | My child becomes sad when other children are sad; My child gets upset when another person is acting upset; My child cries or gets upset when seeing another child cry; My child becomes nervous when other children around them are nervous; My child can continue to feel okay even if people around are upset. | My child seems to react to the moods of people around them. |
| MES (Suzuki & Kino, 2008) |  | Even if my friend is troubled, I cannot share his troubles. | My feelings are prone to be influenced by others; I am not prone to being influenced by others’ emotions. |
| TEQ (Spreng et al., 2009) | When someone else is feeling excited, I tend to get excited too; I remain unaffected when someone close to me is happy. |  | I find that I am “in tune” with other people’s moods. |
|  |  |  | *(continued)* |
| Table S1.2 (continued) |  |  |  |
| Measure | Items addressing Positive SEC | Items addressing negative SEC | Items addressing SEC with no clear valence |
| EmQue (Rieffe et al., 2010) | When my child sees other children laughing, he/she starts laughing too. | When another child cries, my child gets upset too; My child also needs to be comforted when another child is in pain; When another child is upset, my child needs to be comforted too. When another child gets frightened, my child freezes or starts to cry; When other children argue, my child gets upset. |  |
| SES (Shen, 2010) |  |  | I can feel the character’s emotions. |
| QCAE (Reniers et al., 2011) | I am happy when I am with a cheerful group and sad when the others are gloom. | It affects me very much when one of my friends seems upset; I get very upset when I see someone cry; I am happy when I am with a cheerful group and sad when the others are gloom; It worries me when others are worrying and panicky; I am inclined to get nervous when others around me seem to be nervous. | I often get emotionally involved with my friends’ problems; People I am with have a strong influence on my mood. |
| VDQ (Grynberg et al., 2012) |  | I strongly feel the distress of the other; I am unsettled by the other’s tears. |  |
| BES-Y (Heynen et al., 2016) |  | My friend’s unhappiness doesn’t make me feel anything. | I get caught up in other people’s feelings easily; I often get swept up in my friend’s feelings. |
|  |  |  | *(continued)* |
| Table S1.2 (continued) |  |  |  |
| Measure | Items addressing Positive SEC | Items addressing negative SEC | Items addressing SEC with no clear valence |
| EEDS (Karlstetter, 2017) | I remain unaffected when someone close to me is happy. |  |  |
| ESE (Olderbak et al., 2014) | I easily feel happy when the people around me feel happy; When I see that my friend is happy about something, I automatically feel happy as well; I am not easily infected by the happiness of other people; I am not easily infected by the surprise of other people; I easily feel surprise when the people around me feel surprise; When I see that my friend is surprised about something, I easily feel surprise as well. | I am not easily infected by the anger of other people; When I see that my friend is angry about something, I easily feel angry as well; I easily feel angry when the people around me feel angry; When I see that my friend is disgusted about something, I easily feel disgust as well; I am not easily infected by the disgust of other people; I easily feel disgust when the people around me feel disgust; I am not easily infected by the fear of other people;  I easily feel scared when the people around me feel scared; When I see that my friend is scared about something, I easily feel scared as well; I easily feel sad when the people around me feel sad; I am not easily infected by the sadness of other people; When I see that my friend is sad about something, I easily feel sad as well. |  |
|  |  |  | *(continued)* |
| Table S1.2 (continued) |  |  |  |
| Measure | Items addressing Positive SEC | Items addressing negative SEC | Items addressing SEC with no clear valence |
| AMES (Vossen et al., 2015) |  | When a friend is angry, I feel angry too; When my friend is sad, I become sad too; When a friend is scared, I feel afraid; When people around me are nervous, I become nervous too. |  |
| BES-A (Carré et al., 2013) |  | I don’t become sad when I see other people crying; Seeing a person who has been angered has no effect on my feelings; I tend to feel scared when I am with friends who are afraid; My friend’s unhappiness doesn’t make me feel anything. | My friends’ emotions don’t affect me much; I get caught up in other people’s feelings easily; I often get swept up in my friends’ feelings. |
| EI (Jordan et al., 2016) | If I see someone who is excited, I will feel excited myself;  If I see a video of a Baby smiling, I find myself smiling. |  | I sometimes find myself feeling the emotions of the people around me, even if I don’t try to feel what they’re feeling. |
| ECQ (Batchelder et al., 2017) | I am happy when I am with a cheerful group and sad when others are gloom. | It affects me very much when one of my friends is upset; I get very upset when I see someone cry; I am happy when I am with a cheerful group and sad when others are gloom; It worries me when others are worrying and panicky. | The people I am with have a strong influence on my mood;  I tend to get emotionally involved with a friend’s problems;  I can tune into how someone feels rapidly and intuitively; I’m sensitive to the feelings of others. |
|  |  |  | *(continued)* |
| Table S1.2 (continued) |  |  |  |
| Measure | Items addressing Positive SEC | Items addressing negative SEC | Items addressing SEC with no clear valence |
| MEQC (Richaud et al., 2017) |  | When I see someone crying who I do not know, I feel like crying; When I am with someone who is sad, it makes me feel sad too. |  |
| EES (Innamorati et al., 2019) | When I see another person is excited because of something that happened to him/her, I feel excited myself. | While I see a friend crying, I feel myself getting teary-eyed. | Those who know me tell me that I am very affected by the emotions of others. |
| PES (Light et al., 2019) | I easily get excited when those around me are lively and happy; I also feel good when someone I know feels good; It often makes me feel good to see the people around me smiling; I can’t help but smile when my friends smile at me; I can’t stop myself from laughing when others are doing so;  I find that other people’s happiness easily rubs off on me. |  |  |
