# Supplementary material for: Susceptibility to positive versus negative emotional contagion: First evidence on their distinction using a balanced self-report measure
Source: PLoS One. 2024 May 14;19(5):e0302890. doi: 10.1371/journal.pone.0302890 (PMC11093349; doi:10.1371/journal.pone.0302890)
Supplement: S1 Table — (DOCX) [file pone.0302890.s002.docx]

**S2 Table. German items and related items in existing scales.**

| English Items | | German Items | References of Similarly Worded Items |
| --- | --- | --- | --- |
| Positive SEC | |  |  |
|  | PSEC1: It cheers me up to be around a jolly person. | Es heitert mich auf, mit einer vergnügten Person zusammen zu sein. | (self-developed) |
|  | PSEC2: It fills me with joy to be around happy people. | Es erfüllt mich mit frohen Gedanken, wenn ich mit frohen Menschen zusammen bin. | Doherty (1997); Caruso & Mayer (1998); Reniers et al. (2011) |
|  | PSEC3: I let myself be infected by someone’s enthusiasm. | Ich lasse mich davon anstecken, wenn jemand Begeisterung zeigt. | Caruso & Mayer (1998); Spreng et al. (2009); Jordan et al. (2016); Innamorati et al. (2019) |
|  | PSEC4: I get cheerful when I am surrounded by cheerful people. | Ich werde selbst fröhlich, wenn ich von fröhlichen Menschen umgeben bin. | (self-developed) |
| Negative SEC | |  |  |
|  | NSEC1: I get nervous when others around me are nervous. | Ich werde selbst nervös, wenn Andere um mich herum nervös sind. | Mehrabian & Epstein (1972); Garton & Gringart (2005); Dadds et al. (2008); Reniers et al. (2011); Vossen et al. (2015) |
|  | NSEC2: I get angry when I am surrounded by enraged people. | Ich werde selbst ärgerlich, wenn ich von wütenden Menschen umgeben bin. | Vossen et al. (2015) |
|  | NSEC3: I tense up when I hear people fighting. | Ich werde selbst angespannt, wenn ich Leute streiten höre. | Doherty (1997); Rieffe et al. (2010) |
|  | NSEC4: I get stressed when I am around stressed people. | Ich werde selbst angespannt, wenn ich mit gestressten Leuten zusammen bin. | Doherty (1997) |
|  | | | |
