# Supplementary material for: Susceptibility to positive versus negative emotional contagion: First evidence on their distinction using a balanced self-report measure
Source: PLoS One. 2024 May 14;19(5):e0302890. doi: 10.1371/journal.pone.0302890 (PMC11093349; doi:10.1371/journal.pone.0302890)
Supplement: S2 Table — (DOCX) [file pone.0302890.s003.docx]

### **S3 Table. Factor matrix of all 10 items initially tested in Study 1.**

|  | Factor 1 | Factor 2 |
| --- | --- | --- |
| It cheers me up to be around a jolly person. | .69 |  |
| It fills me with joy to be around happy people. | .83 |  |
| I get carried away when someone is euphoric. | .59 | .12 |
| I let myself be infected by someone’s enthusiasm. | .76 |  |
| I get cheerful when I am surrounded by cheerful people. | .71 | .17 |
| I depresses me when people around me are gloomy. | .10 | .49 |
| I get nervous when others around me are nervous. |  | .65 |
| I get angry when I am surrounded by enraged people. |  | .51 |
| I tense up when I hear people fighting. |  | .68 |
| I get stressed when I am around stressed people. |  | .85 |
| *Note*. Only factor loading >.1 are displayed | | |
