# Supplementary material for: Susceptibility to positive versus negative emotional contagion: First evidence on their distinction using a balanced self-report measure
Source: PLoS One. 2024 May 14;19(5):e0302890. doi: 10.1371/journal.pone.0302890 (PMC11093349; doi:10.1371/journal.pone.0302890)
Supplement: S3 Table — (DOCX) [file pone.0302890.s004.docx]

**S4 Table. Bivariate correlations of all measures with social desirability.**

|  | KSEG  Positive Qualities | |  | KSEG  Negative Qualities | |
| --- | --- | --- | --- | --- | --- |
|  | *r* | *p* |  | *r* | *p* |
| Positive SEC | .20 | .0017 |  | -.16 | .0098 |
| Negative SEC | -.35 | .0000 |  | .19 | .0033 |
| AMES Cognitive Empathy | .28 | .0000 |  | -.12 | .0700 |
| AMES Affective Empathy | -.22 | .0006 |  | .09 | .1774 |
| AMES Sympathy | .18 | .0053 |  | -.20 | .0012 |
| IRI Perspective Taking | .46 | .0000 |  | -.13 | .0410 |
| IRI Fantasy | .15 | .0210 |  | -.01 | .8318 |
| IRI Empathic Concern | .32 | .0000 |  | -.22 | .0006 |
| IRI Personal Distress | -.31 | .0000 |  | .09 | .1848 |
| PANAS Positive Affect | .30 | .0000 |  | .30 | .0000 |
| PANAS Negative Affect | -.11 | .0835 |  | .08 | .2259 |
| BFI2 Sociability | .15 | .0164 |  | .00 | .9866 |
| BFI2 Assertiveness | .26 | .0000 |  | .01 | .9066 |
| BFI2 Activity/Energy Level | .27 | .0000 |  | -.06 | .3120 |
| BFI2 Compassion | .32 | .0000 |  | -.34 | .0000 |
| BFI2 Trust | .23 | .0003 |  | -.24 | .0002 |
| BFI2 Respectfulness | .33 | .0000 |  | -.40 | .0000 |
| BFI2 Emotional Volatility | -.46 | .0000 |  | .32 | .0000 |
| BFI2 Depression | -.28 | .0000 |  | .18 | .0039 |
| BFI2 Anxiety | -.32 | .0000 |  | .16 | .0135 |
| EDS Depression | -.20 | .0015 |  | .14 | .0272 |
| GAD7 Anxiety | -.23 | .0003 |  | .19 | .0024 |
| PSS Stress | -.20 | .0016 |  | .22 | .0004 |
| CHIPS Physical Symptoms | -.22 | .0006 |  | .20 | .0018 |
| SWLS Life Satisfaction | .24 | .0002 |  | -.14 | .0293 |
|  | | | | | |
