# Supplementary material for: Susceptibility to positive versus negative emotional contagion: First evidence on their distinction using a balanced self-report measure
Source: PLoS One. 2024 May 14;19(5):e0302890. doi: 10.1371/journal.pone.0302890 (PMC11093349; doi:10.1371/journal.pone.0302890)
Supplement: S4 Table — (DOCX) [file pone.0302890.s005.docx]

**S5 Table. Zero-order correlations of Positive and Negative SEC with all measures.**

|  | | Positive SEC |  | Negative SEC |
| --- | --- | --- | --- | --- |
|  | | *r* (*p*) |  | *r* (*p*) |
| Measures of Empathy | |  |  |  |
|  | IRI Perspective Taking | .26 (< .0001) |  | -.12 (.0536) |
|  | IRI Fantasy | .32 (< .0001) |  | .15 (.0222) |
|  | AMES Affective Empathy | .22 (.0004) |  | .70 (< .0001) |
|  | AMES Cognitive Empathy | .34 (< .0001) |  | .02 (.7734) |
| Measures of Positive/Negative Emotionality and Distress | |  |  |  |
|  | PANAS Positive Affect | .21 (.0009) |  | -.04 (.5063) |
|  | PANAS Negative Affect | -.27 (< .0001) |  | .29 (< .0001) |
|  | IRI Personal Distress | .04 (.5035) |  | .62 (< .0001) |
|  | BFI2 Emotional Volatility | -.10 (.1350) |  | .53 (< .0001) |
|  | BFI2 Depressiveness | -.10 (.1212) |  | .46 (< .0001) |
|  | BFI2 Anxiety | .01 (.8794) |  | .57 (< .0001) |
|  | BFI2 Activity/Energy Level | .39 (< .0001) |  | -.20 (.0020) |
| Measures of Well-being and Mental/Physical Health | |  |  |  |
|  | EDS Depressiveness | -.05 (.4591) |  | .34 (< .0001) |
|  | GAD7 Anxiety | .08 (.2384) |  | .48 (< .0001) |
|  | PSS Stress | -.02 (.7274) |  | .41 (< .0001) |
|  | CHIPS Physical Symptoms | .03 (.6073) |  | .41 (< .0001) |
|  | SWLS Life Satisfaction | .17 (.0079) |  | -.32 (< .0001) |
| Measures of Social/Interpersonal Functioning | |  |  |  |
|  | AMES Sympathy | .38 (< .0001) |  | .15 (.0204) |
|  | IRI Empathic Concern | .46 (< .0001) |  | .13 (.0371) |
|  | BFI2 Sociability | .35 (< .0001) |  | -.18 (.0052) |
|  | BFI2 Assertiveness | .18 (.0038) |  | -.37 (< .0001) |
|  | BFI2 Compassion | .38 (< .0001) |  | -.10 (.1200) |
|  | BFI2 Trust | .28 (< .0001) |  | -.18 (.0043) |
|  | BFI2 Respectfulness | .25 (< .0001) |  | -.29 (< .0001) |
| Measures of Social Desirability | |  |  |  |
|  | Positive Qualities | .20 (.0017) |  | -.35 (< .0001) |
|  | Negative Qualities | -.16 (.0098) |  | .19 (.0033) |
|  | | | | |
