# Supplementary material for: Susceptibility to positive versus negative emotional contagion: First evidence on their distinction using a balanced self-report measure
Source: PLoS One. 2024 May 14;19(5):e0302890. doi: 10.1371/journal.pone.0302890 (PMC11093349; doi:10.1371/journal.pone.0302890)
Supplement: S5 Table — (DOCX) [file pone.0302890.s006.docx]

**S6 Table. Non-parametric Spearman correlations of Positive and Negative SEC with all measures.**

|  | Positive SEC | |  | Negative SEC | |
| --- | --- | --- | --- | --- | --- |
|  | *r* | *p* |  | *r* | *p* |
| AMES Cognitive Empathy | .37 | < .0001 |  | .01 | .8850 |
| AMES Affective Empathy | .21 | .0011 |  | .68 | < .0001 |
| AMES Sympathy | .42 | < .0001 |  | .15 | .0173 |
| IRI Perspective Taking | .25 | < .0001 |  | -.14 | .0343 |
| IRI Fantasy | .31 | < .0001 |  | .19 | .0037 |
| IRI Empathic Concern | .47 | < .0001 |  | .14 | .0307 |
| IRI Personal Distress | .00 | .9719 |  | .59 | < .0001 |
| PANAS Positive Affect | .24 | .0001 |  | -.03 | .6366 |
| PANAS Negative Affect | -.26 | < .0001 |  | .25 | < .0001 |
| BFI2 Sociability | .36 | < .0001 |  | -.15 | .0189 |
| BFI2 Assertiveness | .22 | .0004 |  | -.31 | < .0001 |
| BFI2 Activity/Energy Level | .40 | < .0001 |  | -.18 | .0043 |
| BFI2 Compassion | .38 | < .0001 |  | -.11 | .0947 |
| BFI2 Trust | .29 | < .0001 |  | -.19 | .0022 |
| BFI2 Respectfulness | .30 | < .0001 |  | -.29 | < .0001 |
| BFI2 Emotional Volatility | -.10 | .1005 |  | .48 | < .0001 |
| BFI2 Depression | -.13 | .0456 |  | .43 | < .0001 |
| BFI2 Anxiety | -.02 | .7337 |  | .53 | < .0001 |
| EDS Depression | -.04 | .5009 |  | .32 | < .0001 |
| GAD7 Anxiety | .04 | .5851 |  | .49 | < .0001 |
| PSS Stress | -.04 | .5536 |  | .39 | < .0001 |
| CHIPS Physical Symptoms | .03 | .6024 |  | .39 | < .0001 |
| SWLS Life Satisfaction | .20 | .0013 |  | -.28 | < .0001 |
| KSEG Positive Qualities | .18 | .0038 |  | -.33 | < .0001 |
| KSEG Negative Qualities | -.18 | .0038 |  | .21 | .0010 |
|  | | | | | |
