# Supplementary material for: Susceptibility to positive versus negative emotional contagion: First evidence on their distinction using a balanced self-report measure
Source: PLoS One. 2024 May 14;19(5):e0302890. doi: 10.1371/journal.pone.0302890 (PMC11093349; doi:10.1371/journal.pone.0302890)
Supplement: Attachment — Submitted filename: Response to Reviewers.docx [file pone.0302890.s007.docx]

Response to Reviewers (Manuscript: PONE-D-23-10800)

-

**JOURNAL REQUIREMENTS**

**1. Please ensure that your manuscript meets PLOS ONE's style requirements, including those for file naming. The PLOS ONE style templates can be found at https://journals.plos.org/plosone/s/file?id=wjVg/PLOSOne_formatting_sample_main_body.pdf and https://journals.plos.org/plosone/s/file?id=ba62/PLOSOne_formatting_sample_title_authors_affiliations.pdf**

Author Response: Thank you for pointing our attention to this issue. We ensured that the uploaded files of our resubmission followed those style requirements.

**2. We note that you have referenced (ie. Bewick et al. [5]) which has currently not yet been accepted for publication. Please remove this from your References and amend this to state in the body of your manuscript: (ie “Bewick et al. [Unpublished]”) as detailed online in our guide for authors http://journals.plos.org/plosone/s/submission-guidelines#loc-reference-style**

Author Response: Thank you for bringing this to our attention. However, we are not entirely sure about this feedback. In our manuscript, Bewick et al. was not cited, and our reference [5] was Hatfield et al. (1993). Nevertheless, we checked whether we had cited any not-yet published references and ensured that all our cited references were published and cited correctly in our manuscript.

-

**ADDITIONAL EDITOR COMMENTS**

**The journal Plos One publishes only high-quality work. Therefore, I ask the authors to review the manuscript, following the precise and timely directions of the reviewers. In anticipation, I wish you good work.**

Author Response: Thank you for allowing us to revise and resubmit our manuscript. Below, we describe in detail how we responded to each of the reviewers' comments.

-

**REVIEWER #1**

**The reviewed article has a topic that is relevant and of interest to the public. A minor concern is due to the fact that the sample was recruited 5 years ago (2018), before the pandemic, but it is a consistent sample. The referencing used mostly dates back more than 10 years, which could perhaps be a criticism in the article's discussions.**

Author Response: Thank you for your positive evaluation of our manuscript and for raising these important points. Regarding the sample recruitment date, we added a corresponding section in the discussion section including additional references, which reads as follows:

*Last, while both samples were sufficiently large in terms of statistical power for detecting medium and large effects as well as stability of correlations [111], the generalizability of the findings could still be limited due to our chosen sampling approaches and the fact that recruitment and data collection happened before the COVID-19 pandemic. In Study 1, we used a convenience sample of voluntarily recruited teachers. Therefore, it is possible that only highly motivated and healthy individuals took part in the study. In Study 2, we used an online sample recruited through a crowd-based online platform, also voluntarily but with a monetary incentive. While such crowd-based online samples have been found to be reasonably representative of the general population and to provide high-quality data [112], participants in these samples have been found to report increased levels of depressiveness with up to three times higher levels as compared to prevalence estimations in the general population [113]. Moreover, all data had been collected prior to the onset of the COVID-19 pandemic, which had been shown to have impacted individuals' well-being and mental health in the general population worldwide ([114]) and especially in highly vulnerable subgroups (e.g., [115,116]). Finally, both samples were relatively homogeneous regarding cultural background and educational levels, and the language of all measures in our studies was German. Taken together, more diverse samples, recruited especially during or after the pandemic, are needed to replicate and corroborate our findings, and an English version of our novel self-report measure should be developed and validated in English-speaking populations.*

Regarding the references dating back more than 10 years, we have added the following text to the discussion:

*Third, while research on the process of emotional contagion in human social interaction has seemingly been increasing over the past years, SEC as an individual's disposition has been investigated less frequently in past research. As a result, some of the cited publications in the present work date back more than 10 years. Thus, more current scientific inquiries into SEC and its associations with relevant outcomes are needed, such as the present studies.*

-

**REVIEWER #2**

**Thank you for giving me the opportunity to review the article entitled “Susceptibility to positive versus negative emotional contagion: First evidence on their distinction using a balanced self-report measure”. I appreciated the clarity and accuracy of the article; nevertheless, I have some concerns that I listed below.**

Author Response: Thank you for your generally positive evaluation of our manuscript and for raising a number of very important and constructive comments that helped us to revise the paper. Below, we outline how we responded to each of your comments.

**INTRODUCTION**

**The introduction is rich and well written. Below, some issues:**

**1) The definition and conceptualization of the EC seem to me a little too vague and inconsistent. Authors should clarify this point, according to the literature. Moreover, they should better clarify the distinction between EC and empathy.**

Author Response: Thank you for pointing this out. We agree that it is important to clarify the definition of emotional contagion and its distinction from the concept of empathy, and we also observed that the various definitions found in the existing literature often seem to be overlapping or vague. While revising this manuscript, we made an effort to provide as precise as possible definitions while recognizing the variety of perspectives in the existing literature. The revised section read as follows:

*The idea of emotional contagion has been around since the 18th century [12,13], and it was introduced to scientific fields related to psychology at least 100 years ago [14-17]. Since then, various terms have been used to describe this process, including contagion, transmission, transfer, crossover, resonance, or mirroring [3,7,18-23], and different underlying mechanisms have been proposed [4]. While previous studies have often been unclear and inconsistent in their conceptualizations and definitions [24], emotional contagion can be defined - in agreement with several theorists - as a basic interpersonal process that comprises the mostly automatic and unintentional transmission of emotions from an individual to one or more other individuals resulting in a more or less congruent emotional experience [2,5,7,8].*

*In that sense, it can be delineated clearly from the much broader concept of empathy, which itself has recently been discussed in recent reviews as a rather vague and manyfold umbrella term comprising a range of more precisely defined lower-level concepts (see 24-26], for critical discussions of empathy definitions). In line with these reviews, empathy definitions typically encompass understanding others’ emotions and taking their perspective (i.e., cognitive empathy), sharing someone else’s emotions and feeling prosocial concern, compassion, or sympathy for others (i.e., affective empathy), and sometimes even the behavioral tendencies and reactions resulting thereof as well as accurately recognizing others' emotional states [24-26]. However, this rather diverse collection of processes and capacities has been criticized and discussed as a threat to clear and consistent conceptualizations that are needed for scientific advancement and, instead, it has been suggested to bypass the term empathy in favor of more precise definitions of lower-level constructs, such as emotional contagion (e.g., 24,25).*

*Thus, in contrast to empathy, emotional contagion represents a purely affective response to other individuals' emotional experiences (instead of cognitive role-/perspective-taking), it is based on unintentional, automatic, and mostly unconscious processes (instead of deliberately imagining being in another person's situation), and it does not necessarily involve any behavioral reactions towards others (instead of comforting or helping another person). Most critically, though, emotional contagion does not require to distinguish between one's own emotions and the other person's emotions (instead of an at least minimum level of self-other distinction or awareness of another individual's situation that is postulated as a defining feature of empathy) [24-30]. In other words, emotional contagion means that another individual's emotions become my own emotions, while empathy means understanding and responding to another individual's emotional experiences while distinguishing between my response and the other person's emotions or situation.*

Additionally, to foster a more precise and transparent use of definitions of emotional contagion and SEC, we added the following paragraph in the Discussion section under "Directions for Future Research".

*First, to sustainably overcome the confusion regarding definitions and operationalizations of emotional contagion and SEC in future research and to foster a more consistent use of terms and concepts, we suggest - in line with previous theorists (e.g.,[24]) - that researchers explicitly define and transparently operationalize their view on emotional contagion and/or SEC in future studies.*

**2) The Authors should better discuss the relationship between positive and negative affect. This relationship can vary widely across samples, ranging from near zero (as reported) to strongly negative. Authors should discuss the existing evidence.**

Author Responses: Thank you for bringing this to our attention. We agree that it is important to discuss the existing evidence on the association between positive and negative affect more thoroughly. To this end, we have revised the respective paragraphs as follows, bringing in more evidence from this body of literature:

*Originally, SEC has been conceptualized as a unidimensional and global construct. In the present work, we argue for a more complex perspective on an individual’s SEC. Theoretically, our reasoning is rooted in Watson and Tellegen’s [54] consensual model of positive and negative affect. In contrast to emotion theorists who argued for a specific set of discrete emotions [55-58], Watson and Tellegen proposed a more parsimonious model based on the reanalysis of several datasets with positive and negative affect as two general and relatively independent dimensions of emotional experiences that can be subdivided into specific discrete emotions reflecting the valence of the dimensions, such as happiness or anxiety [54,59-61].*

*Since Watson and Tellegen’s proposal in 1985 [54], a range of empirical studies have provided evidence for this assumption. For example, in a study investigating college students' affect over several class sessions and how it is altered by the feedback on their performance, positive and negative affect were found to be unrelated in each measurement. Moreover, while success feedback increased positive affect, it did not impact students' negative affect, and vice versa ([62]). In addition, several studies investigated certain context factors influencing the relationship between positive and negative affect. For instance, in a study examining the influence of culture and gender on the relationship between positive and negative affect, different correlational patterns were found for the subgroups of American men (weak negative correlation between positive and negative emotions) and women (strong negative correlation) versus Chinese men (weak positive correlation) and women (strong positive correlation), respectively ([63]). Additionally, previous studies examining the influence of experienced stress on this association found that greater levels of stress lead to a stronger negative correlation between positive and negative affect ([64,65]). In studies investigating the relation between positive and negative affect for both state and trait measures, the two dimensions were found to be unrelated at the trait level but showed a significant small negative correlation when considering a shorter time interval ([66,67]). Taken together, the existing evidence suggests that people who frequently and intensively experience positive emotions do not systematically experience elevated or decreased levels of negative emotions. As such, the proposal of positive and negative affect as two separable and -more or less- independent dimensions provides a plausible and promising theoretical framework for our reasoning.*

*In line with this framework, we propose two distinct dimensions of SEC: The SEC of positive emotions (Positive SEC) and the SEC of negative emotions (Negative SEC).*

**3) The Authors should better explain why they don’t expect an overarching construct.**

Author Responses: Thank you for raising this important point. In the present contribution, we propose two distinct dimensions of SEC (Positive and Negative SEC) reflecting the emotional valence of the emotions being transmitted via the process of emotional contagion. The key point of the present work, however, is not that we rule out any covariation between these two dimensions (Positive and Negative SEC) in terms of a potential overarching construct which could explain small amounts of variance. Instead, we propose that the conceptual separation of these two dimensions (even if correlated) is insightful and promising for future research as demonstrated by the diverging correlational patterns with other relevant variables. To clarify this, we revised the respective sentences as follows:

*In line with Watson and Tellegen’s model, we do not necessarily expect an antagonistic relation between Positive and Negative SEC (i.e., a higher Positive SEC does not necessarily lead to a lower Negative SEC). While we do not rule out any covariation between the two dimensions, we do not propose that there is a strong higher-order personal tendency to catch emotions of either valence from other people (resulting in a strong positive correlation between Positive and Negative SEC and explaining most of the variance). Instead, we expect Positive SEC and Negative SEC to be largely independent from one another and linked differentially to variables in several important domains of human functioning, namely emotional experiences, social interaction, and individuals’ mental and physical health.*

**4) The Authors presented an interesting systematic literature review of existing scales that explicitly addressed SEC (S1 Appendix); however, I think that Authors should clarify in the Introduction that there are other instruments which contains item of SEC (Positive and Negative Emotions). The Authors should also specify whether the 7 measures of the individuals’ SES targeted adults, or other age groups.**

Author Responses: Thank you for pointing this out. To clarify this point, we now mention that there are 21 scales focusing on different forms of empathy that contain items resembling emotional contagion and we added the information on the target groups of the scales that explicitly addressed SEC. We revised the respective paragraph which now reads as follows:

*Methodologically, previous research relied on self-report measures to assess an individual’s SEC. Following an extensive and systematic literature review (see supporting information S1 Appendix), we identified seven published scales that explicitly addressed SEC (e.g., the Emotional Contagion Scale [44] and the Emotional Empathic Tendency Scale [7]) and 21 scales focusing on different forms of empathy that contained items resembling emotional contagion. Of the seven scales addressing SEC explicitly, four targeted adults ([7,44-46]), one targeted adults and adolescents ([47]), one specifically targeted parents ([48]), and one targeted children ([49]), respectively. All of the seven instruments have been constructed based on the assumption that the personal tendency to catch other individuals’ emotions through the process of emotional contagion is a global and unidimensional construct irrespective of the valence of the transmitted emotion.*

**5) Why didn’t the Authors target also neutral contagion?**

Author Responses: Thank you for bringing up this intriguing question. As elaborated in the introduction, the conceptualization of emotional contagion in the present work implies that a certain emotion gets transmitted from an individual to one or more other individuals. For that contagion process to be instigated, the emotional experience in the "sender" likely has to reach at least some minimal level of intensity and, thus, go beyond a neutral emotional state. Otherwise, one would not even consider the respective emotion to be present in the "sender," which is a prerequisite for the process of emotional contagion. Thus, based on this conceptualization, we believe that such an emotionally neutral state in the "sender" would not be transmitted to another individual in the sense of emotional contagion.

**6) The Authors should better clarify why in the literature there is a focus on SEC of negative emotions.**

Author Responses: Thank you for raising this point. To clarify this, we added detailed information on the number of items that focused on SEC and the relative share of these items that focused on negative emotions, positive emotions, or no explicit emotional valence, separately for all scales that contained items resembling SEC as well as the scales focusing explicitly on SEC. We revised the respective paragraph as follows:

*Within all scales containing items on an individual's SEC, we identified 132 items that focused directly on being susceptible to emotional contagion in their wording. Conspicuously, most of these items focus on negative emotions (54%) as compared to items focusing on positive emotions (26%) or items with no explicit emotional valence (21%). Even more so, within the seven scales addressing SEC explicitly, we identified 50 items focusing directly on SEC, of which 60% addressed negative emotions, 24% addressed positive emotions, and 16% did not make the emotional valence explicit. Taken together, existing scales - and especially the ones explicitly addressing SEC which represent the basis of most previous literature on SEC - seem to be biased towards an individual’s tendency to catch negative emotions and there seems to be a lack of more balanced measures to assess both the SEC of positive and negative emotions.*

**METHOD**

**1) In the S2 Appendix, the Authors presented the items of their scale. It is unclear to me whether item PSEC1, PSEC4, NSEC4 have been newly developed by Authors. Why did they develop a new item for SEC for Negative Emotions? If I understood well, there are already existing validated tools to assess SEC for Negative Emotions. For adapted items, Authors should clarify the translation process. In which way, did the Authors assess content validity?**

Author Responses: Thank you for pointing this out. The items PSEC1 and PSEC4 have been developed by the authors without any clear reference to existing items (given the lack of Positive SEC items in existing measures) in order to cover the concept of Positive SEC more broadly. For NSEC4, there is indeed a highly similarly worded item in one of the existing self-report scales, so the label “self-developed” was not fully adequate here. To rectify this, we have added a note (i.e., "self-developed") in the table in supplement S2 for the items PSEC1 and PSEC4 as well as a reference to the published scale for NSEC4, respectively.

Regarding content validity and the translation process of the adapted items, we have transferred them to German by means of translation-back-translation by two bilingual experts who reached a consent in a committee approach in case of discrepancies in their translations. Accordingly, we revised the respective sentences as follows:

*The selected items were all related to Positive SEC or Negative SEC in their content and wording and were either taken from published German translations or carefully transferred to the German language by means of translation-back-translation by two bilingual experts who reached a consent in a committee approach in case of alternative translations (see supporting information S2 Table).*

**2) What does it mean that the participants have obtained German A levels? The Authors should clarify for a non- German audience.**

Author Responses: Thank you for pointing this out. We have revised the respective sentences as follows:

*The sample of Study 1 consisted of N = 257 teachers (70.4% female; no answer, 1.2%) from more than 40 schools in different German states (Bavaria, 81.3%; Baden-Wurttemberg, 11.3%; other, 6.6%; no answer, 0.8%). Their age ranged from 26 to 64 years (M = 41.8; SD = 10.9; no answer, 2.7%) and 96.9% had obtained the German "Abitur", a qualification level granted to students after usually 12 to 13 years after passing their final exams at the end of secondary education in Germany giving them access to academic higher education (no answer, 1.2%).*

*The sample of Study 2 consisted of N = 247 participants (48.6 % female) that were recruited in 2018 via Clickworker (www.clickworker.com), a German online recruiting service similar to Amazon MTurk (www.mturk.com). Their age ranged from 18 to 75 years (M = 39.9 years; SD = 12.8 years), 55.9 % had obtained the German "Abitur", and 38.5 % indicated to be married.*

**3) It is not clear to me the rate of nonrespondents for Study 1 and Study 2.**

Author Responses: Thank you for pointing this out. Of all questionnaires that were sent out in Study 1, 50.5% were returned to us fully filled out; resulting in a non-responder rate of 49.5%. To clarify this point, we revised the respective sentence as follows:

*Paper-pencil questionnaires were sent out to all participants, with a response rate of 53.2 % which exceeds response rates of previous paper-pencil studies in this specific population [78,79].*

In Study 2, it is more difficult to calculate or estimate a precise non-responder rate due to the fact, that Clickworker does not provide information on how many individuals actually saw our participation offer on the Clickworker website but did not fill out the survey afterwards. In the soscisurvey tool that was used to collect the online survey data, the survey was filled out by all participants that started the survey (i.e., a completion rate of 100%). To clarify this point, we revised the respective sentences as follows:

*While all participants who started to fill out the online questionnaire also completed it (completion rate = 100%), three participants were excluded prior to any further data processing, as preregistered, because they filled out the questionnaire unrealistically fast (i.e., in under five minutes).*

**4) The Authors should give more information about recruitment in Study 1. Was it part of a larger study? How many schools were involved? How did the Authors contact schools?**

Author Responses: Thank you for raising this important point. We revised the respective sentences as follows:

*Study 1 was part of a larger research project ([73]; see [74] for information on the overarching project) and designed to provide first evidence on the psychometric quality of our novel scales, their statistical separability, and their proposed differential relations with a relevant outcome variable, namely emotional exhaustion being one core component of burnout [75].*

*The sample of Study 1 consisted of N = 257 teachers (70.4% female; no answer, 1.2%) from more than 40 schools in different German states (Bavaria, 81.3%; Baden-Wurttemberg, 11.3%; other, 6.6%; no answer, 0.8%).*

*All participants were recruited voluntarily in 2017 through convenience sampling and direct contact with the schools or the teachers (i.e., schools and teachers were contacted via email, telephone, and written informational letters) before giving their informed consent.*

**5) Cronbach’s alpha and McDonald’s omega for the scale of positive qualities of social desirability are low. Please clarify.**

Author Responses: Thank you for pointing this out. While we agree that the internal consistency of the social desirability scale/factor "KSE-G Positive Qualities" is rather low (alpha = .62, omega = .64), there are two possible reasons for this.

To assess social desirability tendencies more economically, the two KSE-G scales/factors have been developed as very short self-report scales and entail only three items, each in contrast to other social desirability scales comprising up to 33 items (e.g., Marlowe-Crown Social Desirability Scale; Crowne & Marlowe, 1960). Further, the three items in the "KSE-G Positive Qualities" scale/factor are rather diverse and heterogeneous and describe three very different behaviors or reaction tendencies to assess the heterogeneous tendency to exaggerate one's positive qualities:

- "In an argument, I always remain objective and stick to the facts."
- "Even if I am feeling stressed, I am always friendly and polite to others."
- "When talking to someone I always listen carefully to what the other person says."

Given that longer scales and homogeneous constructs generally tend to produce greater internal consistency values (Field et al., 2012), it is not surprising that a short and heterogeneous scale struggles to achieve high internal consistency scores. Accordingly, a study comparing several short forms of a social desirability scale, these short versions achieved very low internal consistency scores of .44 to .45 (Barger, 2002). However, Field and colleagues (Field et al., 2012) and Kline (1999) argue that low alpha values can still be acceptable in the case of heterogeneous and diverse constructs, if the items describe valid indicators of the construct being measures (i.e., exaggerating positive qualities). Overall, it seems to be a common dilemma or trade-off between a short and economic length of a scale, its ability to assess a heterogeneous and diverse construct, and its internal consistency.

To deal with this issue, we reported both partial correlations while controlling for social desirability tendencies (in the manuscript) as well as bivariate correlations without any control variables (in supplement S5). To make a comparison of these two tables more accessible, we reformatted the table in supplement S5 using the same sub-headings as in Table 6 in the manuscript.

References:

- Barger S. D. (2002). The Marlowe-Crowne affair: short forms, psychometric structure, and social desirability. Journal of personality assessment, 79(2), 286–305. https://doi.org/10.1207/S15327752JPA7902_11
- Crowne, D. P., & Marlowe, D. (1960). A new scale of social desirability independent of psychopathology. Journal of consulting psychology, 24, 349–354. https://doi.org/10.1037/h0047358
- Field A, Miles J, Field Z. Discovering statistics using R. SAGE; 2012.
- Kline, P. (1999). The handbook of psychological testing (2nd ed.). London: Routledge.
