# Supplementary material for: Susceptibility to positive versus negative emotional contagion: First evidence on their distinction using a balanced self-report measure
Source: PLoS One. 2024 May 14;19(5):e0302890. doi: 10.1371/journal.pone.0302890 (PMC11093349; doi:10.1371/journal.pone.0302890)
Supplement: Attachment — Submitted filename: Response to Reviewers.docx [file pone.0302890.s008.docx]

**Response to Reviewers (Manuscript: PONE-D-23-10800R1)**

*Thank you for allowing us to revise and resubmit our manuscript again. Below, we describe in detail how we responded to the reviewer's comments.*

**Review Comments to the Author**

**REVIEWER #3**

**The manuscript is solid and rigorous in its scientific content. The data has been well analyzed and the conclusions are clear and relevant. The sample size is appropriate for studies of this nature. However, I have some minor suggestions that I detail below:**

*Author Response: Thank you for your positive evaluation of our manuscript and for raising these important points. Below, we outline how we responded to each of your comments.*

**1. The authors explain that the sample of Study 1 consisted of 257 teachers (line 241, page 12). A few lines below (lines 255 and 256), they mention that the questionnaires were sent to all teachers with a response rate of 53.2%. This could give the mistaken idea that the original sample was 257 teachers which was reduced by almost 50%. Were the questionnaires sent to a larger group, corresponding to a broader project (line 230) and only 257 participants responded? Clarifying this point in this section would be beneficial.**

*Author Response: Thank you for pointing this out. We agree that it is important to clarify the explanation of the response rate and the final sample size. The revised section reads as follows (in the "Materials and Methods" section of Study 1 under "Sample and Procedure"):*

All participants were recruited voluntarily in 2017 through convenience sampling and direct contact with the schools or the teachers (i.e., schools and teachers were contacted via email, telephone, and written informational letters). Overall, more than 480 paper-pencil questionnaires were sent out, with a response rate of 53.2 % resulting in our final sample size and exceeding response rates of previous paper-pencil studies in this specific population [78,79]. No incentives were given for participation and no identifiers that could link individual participants to their results were obtained. Thus, all analyses were conducted on anonymous data. All participants gave their informed consent and the study was conducted in accordance with the ethical standards of the APA and the Declaration of Helsinki. The research project received a formal waiver of ethical approval by the ethics review board of the first author’s institution.

**2. Regarding tables preparation, following common practice, it is suggested to avoid including columns that only contain constant values (e.g. “Possible range” and “Normality” in Tables 1 and 4). Such information could be simply mentioned in a table footnote.**

*Author Responses: Thank you for bringing this to our attention. We agree that the Tables 1 and 4 would benefit from omitting these two categories entailing constant values and mentioning the respective information in table notes. The table note that was added to Table 1 reads as follows:*

*Note.* Following EFA, two items were excluded based on their factor loadings and/or cross-loadings resulting in 4 items per scale (see EFA results for detailed information). The response scale of all items ranged from 1-5 and, as tested using Shapiro-Wilks tests, the collected data showed deviations from normality for all items.

*The table note that was added to Table 4 reads as follows:*

*Note.* The response scale of all items ranged from 1-5 and, as tested using Shapiro-Wilks tests, the collected data showed deviations from normality for all items.

**3. On the other hand, it is expected that the explanations of the results precede table presentation. This criterion seems not to be met concerning Table 1. This shows 4 items for each scale, when it had previously been explained that the scales contained 5 items each. The reader may be confused until reaching the explanation offered below, on line 328. It would be good to correct this matter.**

*Author Responses: Thank you for pointing this out. We added this important information in the table note that was added to Table 1. The revised note reads as follows:*

*Note.* Following EFA, two items were excluded based on their factor loadings and/or cross-loadings resulting in 4 items per scale (see EFA results for detailed information). The response scale of all items ranged from 1-5 and, as tested using Shapiro-Wilks tests, the collected data showed deviations from normality for all items.

**4. Something similar occurs with Table 2, which includes results from Study 2, not yet been introduced or explained in the text.**

*Author Responses: Thank you for raising this point. We added the following short reference to Study 2 in the sentence in which Table 2 is mentioned for the first time (in the "Results and Discussion" section of Study 1 under "Exploratory Factor Analyses"):*

Based on item content, Factor 1 was labeled “Positive SEC” and Factor 2 was labeled “Negative SEC” (see Table 2 for the factor loadings of the final 8 items in Study 1 and Study 2).

*Additionally, we added the following table note to Table 2 for further clarification:*

*Note.* In Study 1, EFA was conducted to test the assumption of two separate dimensions of SEC. In Study 2, CFA was conducted to replicate the factor structure that was found in Study 1. For both studies, only factor loading >.1 are displayed.

**5. Concerning Study 2, the authors propose that the objective was to investigate the validity and reliability in terms of its psychometric properties, internal consistencies, and factor structure. However, this has already been tested in Study 1. I suggest slightly modifying the objective wording to reflect that the purpose of Study 2 was to “corroborate,” “confirm,” and/or “expand” the evidence of validity and reliability collected in Study 1.**

*Author Responses: Thank you for bringing this to our attention. We agree that a slight modification of the study's objectives would better reflect the purpose of Study 2. We revised the respective section as follows:*

In Study 2, we had two primary goals: First, we aimed to corroborate and expand the evidence on our novel scales' validity and reliability.

**6. It would also be recommended that the internal consistency values of the scales used to study the external validity of the SEC (empathy, positive/negative emotionality, etc.) be reported in the Measures section and not in the Results.**

*Author Responses: Thank you for bringing this to our attention. We moved the internal consistency values to the Measures section. Additionally, we decided to keep the internal consistency values in Table 5 as well, in order to make it easier for the reader to compare all psychometric properties for each scale and across all scales within the same table. The revised section reads as follows (in the "Materials and Methods" section of Study 2 under "Measures"):*

Measures of Empathy: To assess different components of empathy, we used scales from the Interpersonal Reactivity Index (IRI; [86,95]) and the Adolescent Measure of Empathy and Sympathy (AMES; [87]), which has been validated in adult samples as well [96]. The IRI Perspective-Taking scale (Cronbach’s alpha α = .76; McDonald’s omega total ω = .81) measures an individual’s tendency to adopt another's perspective to find out what another person might be thinking, thus, representing the cognitive empathy component. The IRI Fantasy scale (α = .75; ω = .81) measures an individual’s tendency to imaginatively transpose oneself into the feelings and actions of fantasy characters. The AMES Affective Empathy scale (α = .81; ω = .84) aims to measure the tendency to vicariously experience another person’s emotions which is quite similar to the concept of emotional contagion. The AMES Cognitive Empathy scale (α = .82; ω = .86) aims to measure an individual’s capacity to cognitively understand another person’s emotions.

Measures of Positive/Negative Emotionality and Distress: To assess different aspects of individuals’ emotionality and distress, we used scales of the IRI and the Big-Five-Inventory 2 (BFI2; [88,97]) as well as the Positive and Negative Affect Schedule (PANAS; [59,98]). The IRI Personal Distress scale (α = .76; ω = .80) measures an individual’s self-oriented tendency to experience distress and anxiety in highly emotional interpersonal situations, such as emergencies. Within the BFI2, the Big Five personality traits can be further subdivided in more differentiated facets. From the Negative Emotionality trait (formerly Neuroticism), we used the BFI2 Emotional Volatility (α = .75; ω = .80), BFI2 Depressiveness (α = .83; ω = .87), and BFI2 Anxiety (α = .69; ω = .77) facets and the BFI2 Activity/Energy Level (α = .73; ω = .79) facet from the Extraversion trait, respectively. The PANAS scales (Positive Affect and Negative Affect) assess an individual’s general experience of positive (α = .88; ω = .91) and negative affect (α = .89; ω = .92) and consist of adjectives describing discrete emotional experiences that are either positive or negative in their valence (instruction: “How do you feel in general?”).

Measures of Well-being and Mental/Physical Health: We used the Edinburgh Depression Scale (EDS; [89,99,100]) to assess current depressive symptoms (α = .87; ω = .90), the General Anxiety Disorder 7 questionnaire (GAD-7; [90,101]) to assess current symptoms of general anxiety (α = .88; ω = .91), and the Perceived Stress Scale (PSS-10; [91,102]) to assess individuals’ current levels of stress (α = .87; ω = .90). To assess individuals’ current physical symptoms (α = .80; ω = .86), we used a short version of the Cohen-Hoberman-Inventory of Physical Symptoms (CHIPS; [92,103]) and to assess individuals’ satisfaction with their lives and current living conditions (α = .93; ω = .94), we used the Satisfaction with Life Scale (SWLS; [93]).

Measures of Social/Interpersonal Functioning: To assess different aspects of individuals’ social and interpersonal functioning, we used scales of the IRI, the AMES, and specific facets of BFI2 personality traits. The AMES Sympathy subscale (α = .68; ω = .77) aims to measure an individual’s tendency to feel concerned or sorrow for another person. The IRI Empathic Concern subscale (α = .69; ω = .71) aims to measure an individual’s tendency to feel concern and sympathy towards others. From the personality trait extraversion, we used the BFI2 Sociability (α = .88; ω = .82) and BFI2 Assertiveness (α = .78; ω = .80) facets and the BFI2 Compassion (α = .64; ω = .71), BFI2 Trust (α = .61; ω = .73), and BFI2 Respectfulness (α = .73; ω = .80) facets from the personality trait Agreeableness, respectively.

Social Desirability: Given that self-reported health-related and clinical psychological variables have been reported to be associated with social desirability ([94]), we decided to assess the participants' social desirability response tendencies. To this end, we used a short German measure (KSE-G; [94]) of the tendency to either exaggerate one’s positive qualities (α = .62; ω = .64) or to conceal negative qualities (α = .70; ω = .72)

**7. On the other hand, I think it might be useful to introduce somewhere in the manuscript, before presenting the desirability scale, a brief justification of why it was considered important to include it.**

*Author Responses: Thank you for raising this point. We added the following brief justification right before presenting the social desirability scale in the Measures section:*

Social Desirability: Given that self-reported health-related and clinical psychological variables have been reported to be associated with social desirability ([94]), we decided to assess the participants' social desirability response tendencies. To this end, we used a short German measure (KSE-G; [94]) of the tendency to either exaggerate one’s positive qualities (α = .62; ω = .64) or to conceal negative qualities (α = .70; ω = .72)

**8. Finally, perhaps it could be interesting to review whether it is appropriate to use parametric correlations, depending on the compliance (or not) of the normality of the data.**

*Author Responses: Thank you for bringing up this important question. We agree that the choice of parametric correlations in this study should be explained more transparently.*

*First, given that Shapiro-Wilk tests are vulnerable to sample size and tend to become significant more easily in larger samples, it is generally suggested to inspect the distribution of the collected data visually as well as their skewness levels in addition to applying a Shapiro-Wilk test (e.g., Field et al., 2012).*

*Thus, we visually inspected our data using histogram plots. Of all 27 variables, only the variables PANAS negative affect and GAD7 anxiety appeared to be clearly skewed to the right (i.e., positively skewed) based on these plots. This impression was substantiated when looking at their skewness values reported in Table 5, whereas most other variables appeared to be approximately symmetric (with values of zero or close to zero) or only moderately skewed (with values between -.5 and +.5).*

*Second, Pearson correlations have long been argued to be robust against violations of the normality assumption (e.g., Havlicek & Peterson, 1976), especially in larger samples (e.g., due to the central limit theorem).*

*Taken together, we consider the distribution of our variables as sufficiently normal and Pearson correlations as sufficiently robust to use parametric correlations.*

*However, to increase research transparency, we have calculated non-parametric Spearman correlations with positive SEC and negative SEC for all variables and added these correlations as an additional supplement file (see supplement S6).*

*Regarding positive SEC, for example, the correlation with empathic concern changed from .47 to .42 and remained highly significant (p < .0001). The correlation with EDS depressiveness changed from .00 to .-.04 but remained far from significant (i.e., p = .9614/.5009).*

*Regarding negative SEC, for instance, the correlation with the variable BFI2 Trust changed from -.10 to -.19 but did not reach significance (p = .1375/.0022). On the other hand, the correlation with the variable PANAS positive affect changed from -.19 to -.03 and remained far from statistical significance (p = .0027/.6366).*

*In sum, while some correlations are slightly larger or smaller when applying non-parametric correlations, the asymmetric correlational patterns for positive SEC versus negative SEC as empirical foundation of our argument for distinct dimensions of SEC, appears to hold true.*

*To further maximize research transparency, we have added the R-code for these histogram plots as well as the non-parametric Spearman correlations to the R-script "study-02.R" in the supplement and updated the file that is available in our OSF repository.*

*To include these considerations in the manuscript, we have added the following explanation in (in the "Materials and Methods" section of Study 2 under "Statistical Analyses") as well as an additional Table in the supplement (S6) comprising the non-parametric correlations of Positive SEC and Negative SEC with all variables:*

While the distribution of almost all variables were judged as non-normal based on Shapiro-Wilk tests, we still consider the distribution of our variables sufficiently normal based on visual inspection of the collected data (all data visualizations are included in the analysis scripts in the supplement) and the respective skewness levels ([85]), especially in light of the generally assumed robustness of Pearson correlations against violations of the normality assumption (e.g., [110]; see supporting information S6 Table for supplemental non-parametric Spearman correlations of Positive and Negative SEC with all measures.).
